# Face Attractiveness versus Artistic Beauty in Art Portraits: A Behavioral Study

**DOI:** 10.3389/fpsyg.2017.02254

**Published:** 2017-12-22

**Authors:** Katharina Schulz, Gregor U. Hayn-Leichsenring

**Affiliations:** ^1^Psychology of Beauty Group, Institute of Anatomy I, University Hospital Jena, Jena, Germany; ^2^DFG Research Unit Person Perception, Friedrich Schiller University Jena, Jena, Germany; ^3^Neurology Department, University of Pennsylvania, Philadelphia, PA, United States

**Keywords:** attractiveness, beauty, perceptual contrast, gist, art portraits

## Abstract

From art portraits, the observer may derive at least two different hedonic values: The *attractiveness* of the depicted person and the *artistic beauty* of the image that relates to the way of presentation. We argue that *attractiveness* is a property that is predominantly driven by perceptual processes, while the perception of *artistic beauty* is based predominantly on cognitive processing. To test this hypothesis, we conducted two behavioral experiments. In a gist study (Experiment 1), we showed that ratings on *attractiveness* were higher after short-term presentation (50 ms) than after long-term presentation (3000 ms), while the opposite pattern was found for *artistic beauty*. In an experiment on perceptual contrast (Experiment 2), we showed that the perceptual contrast effect was stronger for *attractiveness* than for *artistic beauty.* These results are compatible with our hypothesis that appreciation of *artistic beauty* is cognitively modulated at least in part, while processing of *attractiveness* is predominantly driven perceptually. This dichotomy between cognitive and perceptual processing of different kinds of beauty suggests the participation of different neuronal mechanisms.

## Introduction

In the 2003 drama “Girl with a Pearl Earring”, Jan Vermeer’s patron Pieter Van Ruijven states: “How difficult can it be to paint a beautiful girl?” Nevertheless, he insists that the girl should be painted by Vermeer, whom he considers the most talented painter in Delft at his time. Therefore, he appreciates not only the attractiveness of the depicted girl – a maid named Griet – but also the beauty of the painting itself. Otherwise, anybody could have painted the girl. Consequently, there seem to be two different hedonic values in portrait paintings: the *attractiveness* of the depicted person and the *artistic beauty* of the painting.

Despite the Latin maxim: “De gustibus non est disputandum” (“about taste, one must not discuss”), there has been a large amount of research in the field of empirical aesthetics. Due to importance of one’s physical appearance in social life, it is not surprising that a lot of research is devoted to human (physical) attractiveness. Although there is an individual taste, certain facial properties, such as large eyes, a small chin (Cunningham et al., 1995), symmetry (Rhodes et al., 1998), and averageness of the face (Langlois and Roggman, 1990) are overall preferred. The mentioned preferences are cross-cultural (Rhodes et al., 2001a, 2003) and innate (Slater et al., 1998; Scheib et al., 1999). Generally, men who possess more masculine facial features are considered to be more attractive (Scheib et al., 1999). Some authors argue that women’s preferences for masculine men are not constant but change within the menstrual cycle (Gangestad et al., 2004). When ovulating, women prefer more masculine men. This might imply an evolutionary benefit from the selection of a (masculine) attractive partner. Physical attractiveness is a marker for perceived health and, possibly, also for actual health (Rhodes et al., 2001b). Therefore, appreciation of attractiveness seems to be partly universal, partly innate and evolutionary beneficial. Subsequently, the aesthetic appreciation of human faces appears to be deeply embedded in biological mechanisms. However, cognitively driven processes like knowledge and attitudes also have an influence on attractiveness ratings (Leder et al., 2010).

The perception of aesthetic preferences is not restricted to faces (and other natural objects). Also, cultural objects (artworks in the first place, but also design objects, architecture and fashion objects, amongst others) can be considered beautiful.

The visual arts are strongly associated with the concept of beauty. Over the last centuries, there has been an effort to find objective criteria for beauty in artworks (Fechner, 1876; McManus, 1980; Graham and Field, 2007; Redies, 2007). One reason for the difficulties in this approach is the heterogeneity of the objects referred to as artworks. Therefore, many aspects may or may not have an effect on the aesthetic outcome. Another reason is the lack of an overall definition of beauty. In order to create a research framework, several models of aesthetic appreciation have been established.

In several current models of visual aesthetic experience two processing modes of aesthetic stimuli are postulated (Jacobsen, 2006; Chatterjee and Vartanian, 2014). Redies (2015) describes a *perceptual mode* and a *cognitive mode* of aesthetic processing. On the one hand, *perceptual processing* is mainly based on intrinsic properties of the stimulus. Such properties are processed fast and lead to an experience called ‘aesthetics of perception’. This response is largely influenced by evolution and, thus, a result of a long-time adaptation to our natural environment. On the other hand, *cognitive processing* (leading to ‘aesthetics of cognition’) mainly depends on the context of the stimulus and (subconscious) considerations. Of course, perceptual processes can also have a cognitive component and vice versa (Churchland et al., 1994).

Art portraits are of special interest, because, potentially, both processing modes are activated. The perception of art portraits can be directed toward two different aesthetic aspects (1) the *attractiveness* of the depicted person and (2) the *artistic beauty* of the portrait which refers to the way of presentation of the face in the portrait, i.e., formal aspects of artistic composition. Although ratings on *attractiveness* and the *artistic beauty* are highly correlated in art portraits (Hayn-Leichsenring et al., 2013), it remains unclear how the appreciation of *attractiveness* and the appreciation of *artistic beauty* in art portraits relate to each other. We hypothesize that the evaluation on *attractiveness* is predominantly driven perceptually, whereas, in the evaluation *of artistic beauty*, the influence of cognitive processing is more dominant.

In this study, we focus on a simple question: Can the proposed perceptual differences between *attractiveness* and *artistic beauty* be confirmed by behavioral data on art portraits?

To this aim, we performed two behavioral experiments. (1) We conducted a gist experiment to compare ratings after short-term presentation (STP) with ratings after long-term presentation (LTP). This experiment was designed to investigate which kind of information is utilized for the evaluation *of attractiveness* and *artistic beauty*. (2) We studied the perceptual contrast effect in ratings on *attractiveness* and *artistic beauty*. We speculated that *attractiveness*, which is supposedly more perceptually driven, exhibits a stronger liability to perceptual contrast than *artistic beauty*, which is (supposedly) more cognitively driven.

## Materials and Methods

### Experiment 1: Gist

*Gist* perception is one method to study art portraits with regard to similarities and differences of the appreciation of *attractiveness* and *artistic beauty*. The term *gist* refers to a method of short-term and masked presentation and perception. By means of this method, information deriving from visual stimuli is supposedly filtered (Thielsch and Hirschfeld, 2012). Only the essential visual information or the central idea (namely the “gist”) is perceived. The majority of gist experiments deals with categorization and identification tasks. Previously, a superordinate effect has been demonstrated (Mace et al., 2009; Vanmarcke and Wagemans, 2015). This effect implies that, in comparison to basic object level (e.g., dog), the categorization of subordinate object level (e.g., animal) is faster and more accurate. Furthermore, the categorization of singular objects is faster and more accurate than the categorization of complex natural scenes (Vanmarcke and Wagemans, 2015). Additionally, the surrounding of the object is of utmost importance. In a meta-analysis, Oliva and Torralba (2007) showed that objects presented in a familiar context can be recognized faster.

In aesthetic research, gist experiments have shown that first impressions concerning the visual appeal of actual websites are consistent and accurate even at exposure times less than 500 ms (Lindgaard et al., 2006). At 50 ms the ratings were reliable and a mere exposure effect appeared. Other rating experiments focused on facial attractiveness. Locher et al. (1993) revealed that attractiveness can be assessed within a glance of 100 ms. This finding was replicated by (Olson and Marshuetz, 2005) by means of an exposure time of 14 ms. The participants were able to assess the facial attractiveness of the stimuli quite accurately although they were not even aware of the images they had seen.

Concerning artworks, viewers are able to capture the structural composition and the semantic meaning within 100 ms (Locher et al., 2007). Locher et al. (2007) underlined that even a short glance provides a global impression of the artwork. They found a high correlation between *pleasingness* ratings when stimuli were presented for 100 ms and when stimuli were presented for unlimited time. On average, unlimited *pleasingness* ratings were higher than 100 ms *pleasingness* ratings. This might be explained by “fluency” (Reber et al., 1998). The fluency of an object facilitates processing and, therefore, increases liking. Possibly, in short-time presentations, deliberate (cognitive) processing is limited and perceptual processing predominates. Longer presentation durations might increase perceived fluency and liking (Reber et al., 1998). By means of a Gist experiment we examined the hypothesis that the perception of *artistic beauty* and *attractiveness* is processed differently, respectively.

Here, we tried to replicate the results from Locher et al. (2007), who showed that *artistic beauty* ratings are higher in LTP, and hypothesize that *attractiveness* ratings will be lower in LTP, because *attractiveness* is mediated by perceptually driven processes to a higher degree than *artistic beauty*. Moreover, more visual information may interfere negatively with the first good impression. Therefore, ratings might be decreasing the longer someone watches a face. This would be in accordance with findings of Gerger et al. (2016). In addition, we used other datasets with or without artistic claim. Our goal was to investigate whether there is a restriction of the described rating pattern to portrait paintings or whether there is a pattern in gist ratings for stimuli with artistic claim.

#### Participants

Sixty students (18 to 31 years, *M* = 23.1, 14 male), mainly from medical faculty, participated in this study. Six participants had to be excluded from the original 66 participants due to data collection problems. The participants were divided into three groups (20 participants per group). Both experiments were conducted in accordance with the ethical guidelines of the Declaration of Helsinki and were approved by the ethics committee of Jena University Hospital. All participants gave their written consent prior to the experiment.

#### Stimuli

We used six different datasets *(art portraits, face photographs, landscape paintings, landscape drawings, landscape photographs, and abstract art*,) each of them containing 100 images. *Art portraits* and *landscape paintings* were a subset from the JenAesthetics database (Amirshahi et al., 2013), *face photographs* were random selections from the FACES database (Ebner et al., 2010), landscape drawings and *landscape photographs* were taken from a database established by Redies et al. (2007) and images of *abstract art* were taken from a database established by Mallon et al. (2014).

#### Procedure

**FIGURE 1:**
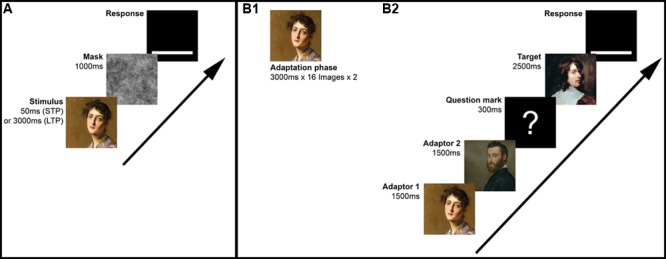
Graphic representation of the experimental designs of Experiment 1 **(A)** and Experiment 2 (**B1**: Adaptation phase, **B2**: Evaluation phase). The displayed images of art portraits in **B1** and **B2** are examples for the adaptation on *artistic beauty* with top-rated images (adaptors) and an intermediate-rated image (target).

The experiment was subdivided in a STP phase and a LTP phase. Due to the length of the experiment, we divided the 60 participants into three groups (20 participants per group). Each group rated only two or three of the datasets in order to prevent a decrease in the participants’ attention. In the first round, the images were presented for 50 ms in random order within the categories. Each image was followed by a random-phase Fourier mask (1000 ms) in order to avoid afterimages. The participants were asked to rate the images by means of a continuous-looking scale appearing on the bottom of the screen. When analyzing the ratings, we decoded the continuous-looking scale (100 steps) to a scale ranging from 0 to 1. After voting on the scale, the next trial started. Therefore, in the first round, 400 images were rated by each participant. In the second round, we presented the same images with the same procedure, but with a presentation time of 3000 ms, a new randomized order and without the Fourier mask. **Figure 1A** shows a graphic representation of the experimental design. We chose this order (first STP, then LTP) to avoid familiarity effects because an LTP in the first round would have led to a better memorization of the stimuli (which we tried to avoid).

The *art portraits* dataset was run in two trials by different participants. The ratings were obtained with regard to the *artistic beauty* (scale ranging from *not beautiful* [German: *nicht schön*] to *beautiful* [*schön*]) and, from another group of participants, with regard to the *attractiveness* of the depicted person (scale ranging from *not attractive* [*nicht attraktiv*] to *attractive* [*attraktiv*]). The meaning of the terms *beautiful* and *attractive* in our study was explained to the participants (*beauty* as a property of the image based on composition, style and use of color and *attractiveness* as a property of the depicted person reflecting their natural/biological appeal). The other datasets were rated by the participants for their pleasantness without any further instruction (scale ranging from *do not like* [*gefällt mir nicht*] to *like* [*gefällt mir*]) with the exception of *face photographs* that were rated for attractiveness (scale ranging from *not attractive* [*nicht attraktiv*] to *attractive* [*attraktiv*]).

The first group of participants rated the datasets *art portraits (*ratings on *artistic beauty), face photographs* and *landscape paintings*. The second group rated the datasets *art portraits (*ratings on *attractiveness)* and *landscape drawings*. The third group rated the datasets *landscape photographs* and *abstract art*.

#### Statistical Data Analysis

We collected the data with the help of a PsychoPy program (Peirce, 2007) that has been created for this purpose by our group. For each image, we computed the response on *artistic beauty* or *attractiveness*. We performed a linear correlation test (Pearson’s *r*) for the ratings after STP and LTP.

Additionally, we performed a Fisher transform to investigate whether the difference in the correlations between STP/LTP *artistic beauty* ratings and STP/LTP *attractiveness* ratings was significant. To this aim, we converted Pearson’s *r* to Fisher’s *z* and computed the confidence interval at 99%.

We determined the mean value of the ratings for the short-term response and the long-term response for each of the image categories. Then, we performed a paired Student’s *t*-test pairing mean measures of each participant for STP with the respective measures for LTP for every category. In order to evaluate the significance for the difference of *art portrait* ratings on *beauty* as compared to *attractiveness*, we conducted a 2 (hedonic value: *beauty* vs. *attractiveness*) × 2 (presentation time: STP vs. LTP) mixed-design ANOVA. Analogously, we performed two additional 2 (category: *landscape paintings* vs. *landscape photographs*/*landscape drawings*) × 2 (presentation time: STP vs. LTP) mixed-design ANOVAs to investigate the influence of presentation time on ratings according to the way of display of landscapes.

#### Results

First, we determined the correlations between the mean ratings of participants after STP and the mean ratings of participants after LTP. We found significant correlations for *beauty* (Pearson’s *r* = 0.569; *p* = 0.006) and *attractiveness* (*r* = 0.923; *p* < 0.001) in *art portraits*. A Fisher transform showed a significant difference of the correlations (*z* = -2.884; *p* = 0.002).

Furthermore, we found significant correlations between the ratings after STP and the ratings after LTP for *face photographs* (*attractiveness*: *r* = 0.879; *p* < 0.001), *landscape paintings* (*r* = 0.901; *p* < 0.001), *landscape drawings* (*r* = 0.660; *p* = 0.002), *landscape photographs* (*r* = 0.770; *p* < 0.001) and *abstract art* (*r* = 0.735; *p* < 0.001).

**FIGURE 2:**
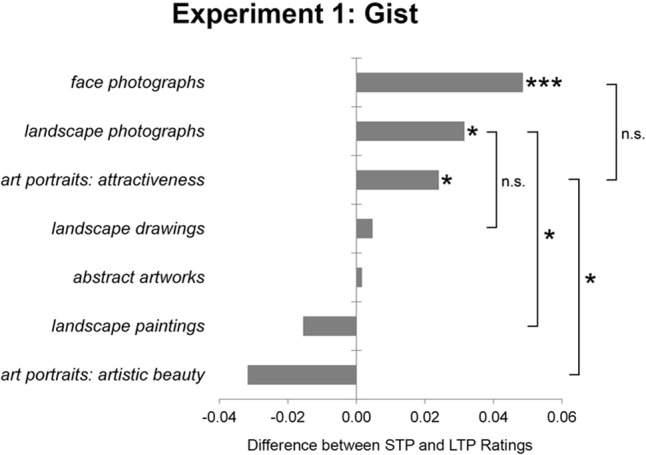
Difference between subjective rating scores (average ratings for short-term presentation STP minus average ratings for long-term presentation LTP) for all tested categories ordered by size. ^∗∗∗^*p* < 0.001, ^∗^*p* < 0.05.

Interestingly, mean values of responses after LTP were significantly lower than mean values for responses after STP for *attractiveness* of *art portraits* (ST: *M* = 0.456; LT: *M* = 0.432; *t*(19) = 1.850; *p* = 0.048), for *liking* of *face photographs* (ST: *M* = 0.491; LT: *M* = 0.443; *t*(19) = 4.055; *p* = 0.001), and for *liking* of *landscape photographs* (ST: *M* = 0.538; LT: *M* = 0.500; *t*(19) = 2.688; *p* = 0.002). For *beauty* in *art portraits*, we found a tendency for higher mean ratings after LTP as compared with mean ratings after STP (ST: *M* = 0.429; LT: *M* = 0.460; *t*(19) = -1.443; *p* = n.s.). See **Figure 2** for detailed results and Supplementary Tables S1, S2 for an analysis in gender.

For art portraits, a 2 × 2 between-subjects ANOVA showed a significant interaction effect of hedonic value (*beauty* vs. *attractiveness*) by presentation time, *F*(1,19) = 5.816; *p* = 0.026, indicating that the effect of presentation time was different between *beauty* and *attractiveness*. *Artistic beauty* ratings were higher after LTP, while *attractiveness* ratings were higher after STP (**Figure 2**).

Analogously, the effect of presentation time was also different between landscape paintings and landscape photographs (2 × 2 between-subjects ANOVA: *F*(1,19) = 9.559; *p* = 0.006). However, another ANOVA revealed that there was no significant effect comparing *landscape paintings* and *landscape drawings* (2x2 between-subjects ANOVA: *F*(1,19) = 0.838; *p* = n.s.).

In conclusion, we found significantly higher values after STP for the photograph categories and *attractiveness* in *art portraits* and no effect or tendencies to higher ratings after LTP for the art categories.

### Experiment 2: Perceptual Contrast

We investigated differences in perceptual contrast between *attractiveness* ratings and *artistic beauty* ratings. Perceptual contrast is an important mechanism of humans to adapt to their changing environment; it is defined as a shift of the evaluation of a stimulus away from the evaluation of the preceding stimulus (Baccus and Meister, 2004). By means of perceptual contrast, perceptual benchmarks and prototypes are continuously renewed. Already 8-year-olds adapt to distorted faces (Anzures et al., 2009) although their fusiform face area is not yet fully developed. Prior studies demonstrated a perceptual contrast effect for gender (Troje et al., 2006), age (Schweinberger et al., 2010), and attractiveness (Rhodes et al., 2003; Hayn-Leichsenring et al., 2013) of faces. Not only attractiveness of faces, but also beauty in abstract paintings is prone to perceptual contrast (Mallon et al., 2014). Hayn-Leichsenring et al. (2013) showed that perceptual contrast occurs for attractiveness in face photographs and art portraits, respectively, as well as for beauty in art portraits. The authors found a high correlation between *attractiveness* and *beauty* ratings. However, they did not investigate possible differences in effect size. Here, we tried to replicate the results and investigated whether the effect sizes of the two hedonic categories differed. We hypothesize that, if *attractiveness* is processed mainly perceptually, there should be a larger perceptual contrast effect for *attractiveness* than for *artistic beauty*, because perceptual mechanisms should be more easily affected by short-time adaptation than cognitive processes.

#### Participants

Forty-four students, mainly from medical faculty, participated in this experiment. Four participants had to be excluded from the experiment due to false instructions. The remaining 40 participants (19 to 29 years, *M* = 23.7, 13 male) were divided into two groups of 20 participants. Every participant took part in only one of the two experiments (*beauty* or *attractiveness*).

#### Stimuli

In Experiment 2, art portraits served as stimuli. We used best-rated and worst-rated images from Experiment 1 (long-term ratings) as adaptors and the intermediate-rated images as test stimuli for the categories *artistic beauty* and *attractiveness*, respectively.

**FIGURE 3:**
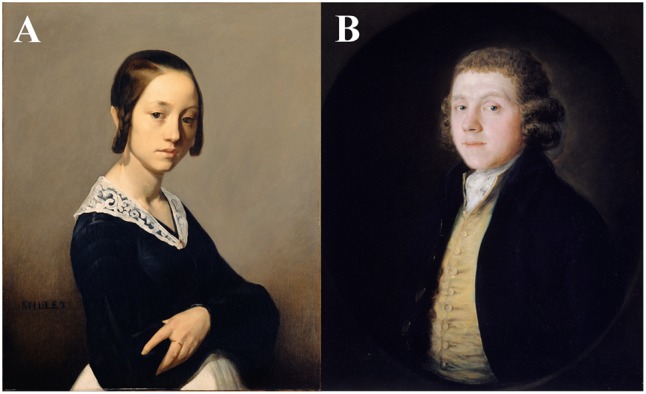
Examples for art portraits: **(A)** “Portrait of Louise-Antoinette Feuardent” by Jean-François Millet (1841). Attractiveness: *Mean* = 0.58; Artistic beauty: *Mean* = 0.56. **(B)** “The Reverend Samuel Kilderbee” by Thomas Gainsborough (ca. 1758). Attractiveness: Mean = 0.24; Artistic beauty: Mean = 0.31. The displayed images were taken from the JenAesthetics database (Amirshahi et al., 2013) and are royalty-free.

We standardized the mean ratings of the adaptors and the stimuli. To this aim, we handpicked the images until the mean values and standard deviations were reasonably similar. Therefore, for *artistic beauty*, we selected sixteen top-rated images (*M* = 0.642, *SD* = 0.055) and sixteen bottom-rated images (*M* = 0.303, *SD* = .038) as adaptors, as well as fifty-two intermediate-rated images (*M* = 0.432, *SD* = 0.055) as target stimuli. An analogous selection was carried out for *attractiveness* (top: *M* = 0.638, *SD* = 0.064; bottom: *M* = 0.297, *SD* = 0.042; intermediate *M* = 0.427, *SD* = 0.065). In Experiment 1, we generated a balanced gender ratio of the depicted persons (100 stimuli, 50 female). After selecting images based on the rating results from the Gist experiment, the gender ratio for Experiment 2 differed between negative and positive adaptors. The *attractive*/*beautiful* adaptors mainly consisted of portraits of younger females whereas the *unattractive*/*not beautiful* adaptors mainly consisted of portraits of older males (see example images in **Figure 3**).

#### Procedure

The experiment consisted of two cycles with two phases: an adaption phase and a following evaluation phase. In the adaptation phase, sixteen art portraits [either *(artistically) beautiful* or *not (artistically) beautiful*] were presented to the participants. The images were randomized and shown twice for 3000 ms. Therefore, the adaptation phase lasted 96 s. Immediately, the evaluation phase followed. The evaluation phase consisted of 52 trials. In each trial, two adaptor images from the same category as the images in the adaptation phase were shown in order to refresh the adaptation (1500 ms each). Then, a question mark was presented on a black screen. Afterward, the target image occurred for 2500 ms. Then, a continuous-looking rating scale, ranging from *not beautiful* [*nicht schön*] to *beautiful* [*schön*], appeared. After the response by mouse click, the next trial started. The 52 target images appeared in random order. After one cycle, the participants took a break and performed a different task for at least 15 min in order to keep transfer effects to a minimum. Subsequently, a second cycle started, arranged similarly to the first, using opposite adaptor images for adaptation. The order of cycles (adaptation to positive or negative stimuli) was counterbalanced across the participants. See **Figure 1B** for a graphic representation of the experimental design.

The Experiment on *attractiveness* ratings was carried out analogously with a scale ranging from *unattractive* [*unattraktiv*] to *attractive* [*attraktiv*]. In the perceptual contrast experiment, 20 participants rated the art portraits for *beauty* (group 1), while 20 other participants rated them for *attractiveness* (group 2).

#### Statistical Data Analysis

Again, we collected the data with the help of a PsychoPy program (Peirce, 2007). For each image, we computed the response after adaptation to *least beautiful* images and after adaptation to *most beautiful* images, as well as after adaptation to *least attractive* persons and after adaptation to *most attractive* persons. We calculated the mean values and compared them with the help of a paired Student’s *t*-test pairing mean measures of each participant after adaptation to *least beautiful* images and after adaptation to *most beautiful* images. The same procedure has been performed for *attractiveness*.

In order to investigate whether the perceptual contrast effects differed in size, we performed a Split Plot ANOVA on the single measures with adaptation condition as within-subject-factor and hedonic value as between-subject-factor

#### Results

**FIGURE 4:**
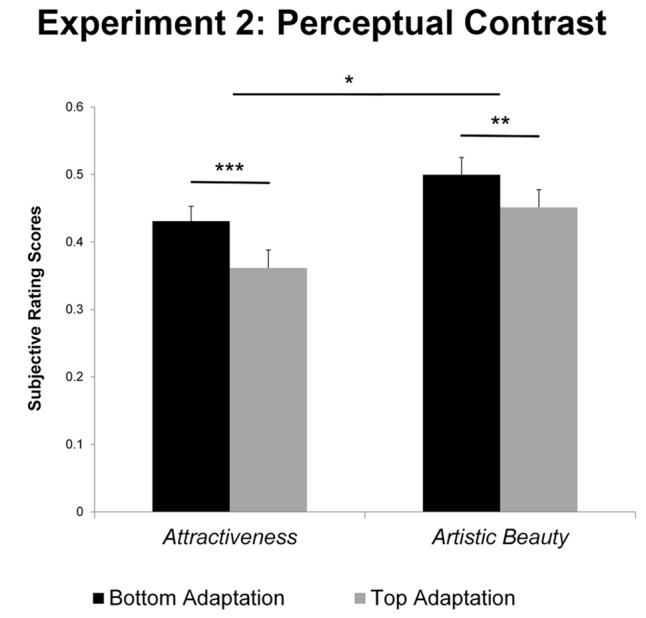
Subjective rating scores after adaptation on top-rated or bottom-rated stimuli for *attractiveness* and *artistic beauty*, respectively. Both differences were significant. However, the perceptual contrast effect on *attractiveness* was significantly larger. Error bars represent standard errors. ^∗∗∗^*p* < 0.001; ^∗∗^*p* < 0.01; ^∗^*p* < 0.05.

We found a significant difference between ratings after adapting to the *most beautiful* images and ratings after adapting to the *least beautiful* images (*most beautiful M* = 0.451, *SD* = 0.036; *least beautiful M* = 0.500, *SD* = 0.026; *difference* = 0.049; *t*-test: *t*(19) = 5.031, *p* < 0.001). We also found significant differences between ratings after adaptation on *most attractive* persons and ratings after adaptation on *least attractive* persons (*most attractive M* = 0.362, *SD* = 0.027; *least attractive M* = 0.431, *SD* = 0.022; *difference* = 0.069; *t*(19) = 3.037, *p* = 0.007). See **Figure 4** for a visual display of the results.

A Split Plot ANOVA confirmed that the interaction between ratings after exposure to different adaptors (*bottom rated vs. top rated*) and hedonic value (*beauty vs. attractiveness*) was significant, *F*(1,2078) = 6.115, *p* < 0.05. Therefore, the effect size of the perceptual contrast differed between *beauty* and *attractiveness*.

## Discussion

We investigated the relation between two different kinds of hedonic values in two behavioral experiments with art portraits as stimuli. These types of visual artworks display the physical beauty of the face, namely *attractiveness*, and the beauty of the image itself, namely *artistic beauty*. Both aspects have different connotations and appearances. Physical *attractiveness* is mainly a natural concept, whereas *artistic beauty* (or the way of representation of the face) is a partly cultural construct. We asked whether this difference is also reflected in the aesthetic appreciation and in the perception modes.

### Gist

Ratings after STP showed a higher correlation with ratings after LTP for *attractiveness* than for *beauty* in *art portraits*. In other words: Based on gist perception ratings, LTP ratings on *attractiveness* are better predictable than LTP ratings on *beauty*. This finding may be seen as an indicator for the different mechanisms of appreciation for these two different hedonic values. Furthermore, the change of the ratings between LTP and STP displayed opposite patterns for *attractiveness* and *artistic beauty* (Experiment 1, **Figure 2**). *Attractiveness* ratings were lower in LTP, whereas *artistic beauty* ratings tended to be lower in STP. This finding is in accordance with results reported by Gerger et al. (2016) who showed that ratings on liking of faces are higher in STP, possibly because more information contributes to a less positive perception of the image. An alternative explanation may be that the perception of fine detail like skin blemishes and wrinkles (represented in high-spatial frequencies), which usually lower the perceived attractiveness (Jones et al., 2004; Samson et al., 2010), is reduced in STP. Low-spatial frequencies have been described as influential for aesthetic judgments in STP (Thielsch and Hirschfeld, 2012) and they can increase the perceived attractiveness of faces (Menzel et al., 2015).

The pattern we found for the ratings on *artistic beauty* is in accordance with the results of Locher et al. (2007). They showed significantly higher ratings on representational (non-portrait) artworks presented for an unlimited time than in STP (100 ms). Cognitive mastering of “mentally challenging” stimuli might contribute to an aesthetic pleasure (Belke et al., 2015). Thus, elaborative mastering – and as a consequence subjective aesthetic pleasure – is supposedly only possible if the observer processes the content and contextual information provided by the portrait. A short glance may be insufficient for this. Therefore, the *artistic beauty* ratings on art portraits were higher in LTP as the participants were provided with the information needed for mastering the art portraits properly. Especially when it comes to modern art, insight is of great importance to the extension of liking (Muth and Carbon, 2013). Therefore, the appreciation of *artistic beauty* might require more time. This is in accordance with Reber et al. (1998) who showed that a longer presentation duration contributes to a higher perceived fluency and – as a consequence – slightly increases the liking of the images.

In order to generalize our results, we performed gist experiments on additional datasets. *Landscape paintings* showed a tendency for a similar rating pattern as *artistic beauty* in *art portraits*. In contrast, *attractiveness* in *face photographs*, as well as *liking* in *landscape photographs* showed a pattern similar to *attractiveness* in *art portraits.* We want to especially emphasize that although artistic displays of faces differ in several properties from photographs (Graham et al., 2014), the pattern of results from the gist experiment is similar for *attractiveness*. Interestingly, on landscape depiction, the artistic category (*landscape paintings*) showed results similar to *artistic beauty* in *art portraits*, possibly because, as mentioned above, mastering of *artistic beauty* requires more time. In contrast, non-artistic *landscape photographs* showed results similar to *attractiveness* in *art portraits*, presumably because the presented photographs displayed mere objects without an obvious cognitive challenge.

These findings of opposite rating patterns may also be explained by the varying exposure frequencies of the depicted objects. Art objects are less common in everyday life and, therefore, the observer is presumably less used to perceiving and judging them. There might not be a prototype for artworks that already exists and facilitates the rating process.

The larger difference between STP ratings and LTP ratings for *attractiveness* in *face photographs* as compared to *attractiveness* in *art portraits* can be explained by the different surroundings of the faces in the images. As Oliva and Torralba (2007) pointed out, scene structure and knowledge of regularities can provide additional information needed for the recognition of the object. This might also be the case for evaluation because, within the presented databases, the *face photographs* studied have a uniform background and the faces are always centered, while this is not the case for *art portraits*. Therefore, recognition (and evaluation) should be easier for *face photographs*.

*Landscape drawings* did not show a difference between ratings after LTP and STP. This may be due to the fact that those images were of very low contrast, which might have led to impaired perception in STP. Presumably, in *abstract artworks*, the colors themselves – mainly perceptually processed properties – influenced *artistic beauty* ratings and, therefore, there was no significant effect.

### Perceptual Contrast

In a second step, we investigated perceptual contrast. Previously, it had been shown that humans adapt to the attractiveness of depicted faces (Rhodes et al., 2003). Hayn-Leichsenring et al. (2013) revealed that not only attractiveness, but also the beauty of the composition of an art portrait underlies adaptive mechanisms. Here, we extend these findings by showing that the magnitude of the perceptual contrast was larger for *attractiveness* than for *artistic beauty* (Experiment 2, **Figure 4**). Perceptual contrast has been described as an evolutionary mechanism to adapt to a constantly changing natural environment. Faces and facial expressions have a strong effect on human interaction and socialization. They play an exceptional role in our visual and processing system. The perceptual contrast effect for *attractiveness* is more prominent than the perceptual contrast effect for *artistic beauty.* This difference might be based on an innate mechanism. Possibly, the short-time adaptation to *attractiveness* is stronger due to its biological nature and/or the more frequent exposure to faces in general. Hence, the brain adapts to a new prototype of faces in order to avoid spending a large capacity of the active brain to the repeating and in most cases unnecessary evaluation of every single face we encounter. In contrast, possibly because of a cognitive overlay, there is a lower perceptual contrast concerning *artistic beauty*. In art portraits, cognitive mastering of challenging art portraits might increase subjective liking (Belke et al., 2015). By scrutinizing the portraits, the observer may be able to recognize the art style and achieve an understanding of the form in the image. These aspects may contribute to an increased pleasantness (in our experiment reflected by the *artistic beauty*) of the portrait. The cognitive processing of an art portrait may cause a cognitive bias during the adaptation process, so that the perceptual contrast is reduced. However, perceptual mechanisms, like the preference for certain global image properties (Redies, 2007) may also play a role for the appreciation of *artistic beauty*. These perceptual mechanisms might explain why, we still found a perceptual contrast effect on *artistic beauty* although it is smaller than the contrast effect on *attractiveness*.

## Limitations

As stimuli, we used images of art portraits and, therefore, we did not show real artworks but smaller representations of artworks. Furthermore, one can assume that the art portraits of the dataset already represent a preselected group of beautiful art portraits, because only *art portraits* of at least to some extent famous artists are part of the Google Art Project database. Hence, the dataset includes mainly relatively beautiful images and, probably, only few images that are less beautiful. Notwithstanding that there is cross-cultural consensus about some features of attractiveness, culture and upbringing may have a large influence on the perception of hedonic value. We did not gather data concerning the participants’ cultural environment and their art expertise, but tried to keep possible biases as stable as possible by testing a homogenous group of participants of similar age (18 to 31), similar cultural background and state of education (primarily German medical students). Additionally, in Experiment 2 (Perceptual Contrast), it is debatable if it is accurate to compare the differences in rating magnitude between scales (for *artistic beauty* and *attractiveness*) by a statistical analysis. The psychological scaling might differ and, therefore, a comparison between the scales is not straightforward. However, we see no other option to compare *artistic beauty* and *attractiveness* ratings.

## Conclusion

*Art portraits* are perceptually challenging. On the one hand, cognitive mastering of image composition and art styles affects the hedonic value that derives from the image (Belke et al., 2015). A longer presentation time allows the processing of more information and thereby enhances the degree of insight into the artwork. Consequently, it leads to a greater hedonic value. We argue that, in art portraits, the appreciation of *artistic beauty* is influenced by cognitive effects to a greater extent than the appreciation of (facial) *attractiveness*.

## Outlook

In future studies, a low-level modulation of face photographs (and possibly art portraits) would allow to investigate, whether the gist results on facial attractiveness are driven by global image properties or higher (conceptual) image features (see Augustin et al., 2008). Furthermore, in order to overcome limitations concerning art expertise, future studies may conduct pre-studies on stimuli selection (see Hayn-Leichsenring, 2017) and, moreover, investigate differences between art experts and lay people (see Leder et al., 2013). Eventually, the evaluations on *artistic beauty* and *attractiveness* could be investigated in brain imaging studies (fMRI) in order to look for differences in neuronal processing.

## Author Contributions

KS planned the study, performed the data collection, helped with the statistical analysis, and wrote the manuscript. GH-L helped planning the study, performed the statistical analysis and wrote the manuscript.

## Conflict of Interest Statement

The authors declare that the research was conducted in the absence of any commercial or financial relationships that could be construed as a potential conflict of interest.
