# Supplementary material for: Face Attractiveness versus Artistic Beauty in Art Portraits: A Behavioral Study
Source: Front Psychol. 2017 Dec 22;8:2254. doi: 10.3389/fpsyg.2017.02254 (PMC5743918; doi:10.3389/fpsyg.2017.02254)
Supplement: Supplementary file 1 [file Table_1.DOCX]

Mean values of short term presentation (STP) and long-term presentation (LTP) subdivided concerning gender.

| **Presentation Time** | *Beauty* | | *Attractiveness* | |
| --- | --- | --- | --- | --- |
|  | **female** | **male** | **female** | **male** |
| **Ultra-rapid** | .471 | .385 | .529 | .379 |
| **Long-term** | .508 | .411 | .498 | .362 |
