# Supplementary material for: Face Attractiveness versus Artistic Beauty in Art Portraits: A Behavioral Study
Source: Front Psychol. 2017 Dec 22;8:2254. doi: 10.3389/fpsyg.2017.02254 (PMC5743918; doi:10.3389/fpsyg.2017.02254)
Supplement: Supplementary file 2 [file Table_2.DOCX]

Mean values of short-term presentation (STP) and long-term presentation (LTP) of *attractiveness* and *artistic beauty* subdivided concerning gender of the depicted person.

| **Adaptors** | **Category** | **female** | **male** |
| --- | --- | --- | --- |
| **Bottom** | *Beauty* | .460 | .522 |
|  | *Attractiveness* | .409 | .452 |
| **Top** | *Beauty* | .412 | .473 |
|  | *Attractiveness* | .342 | .380 |
